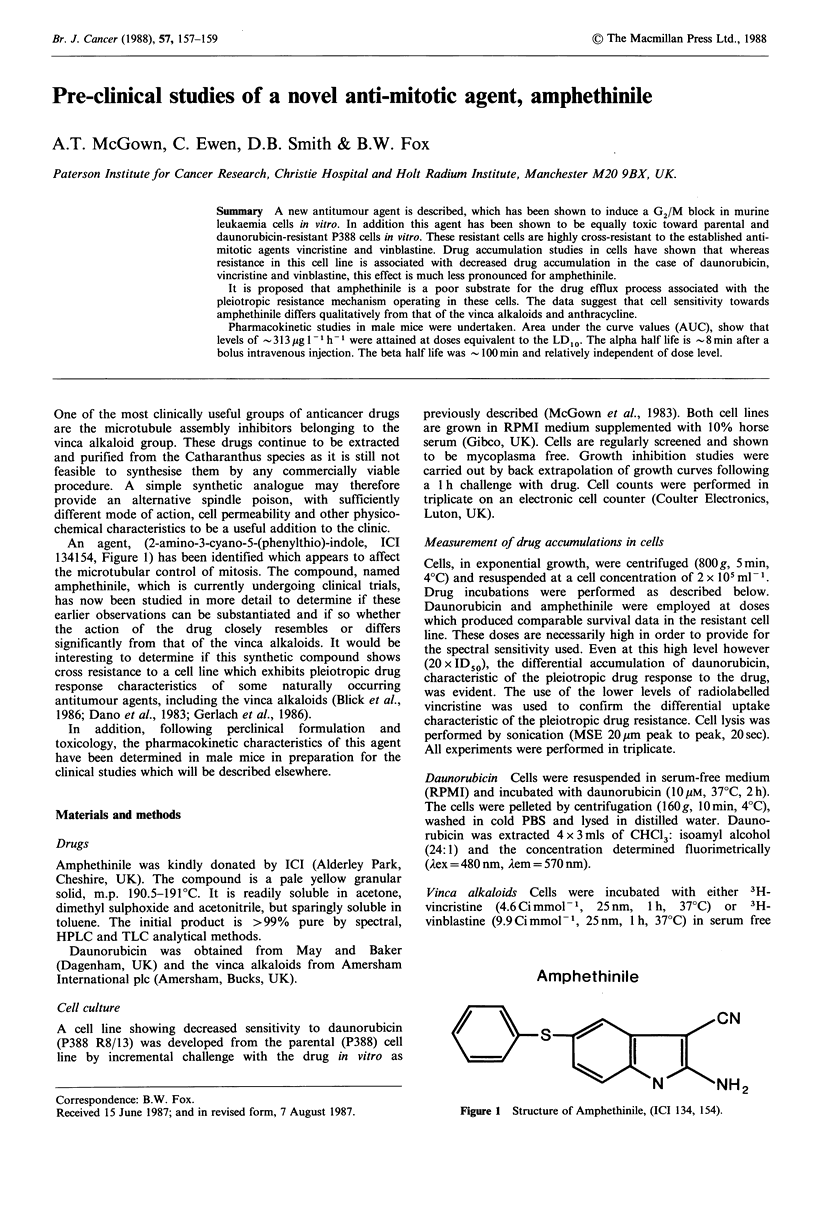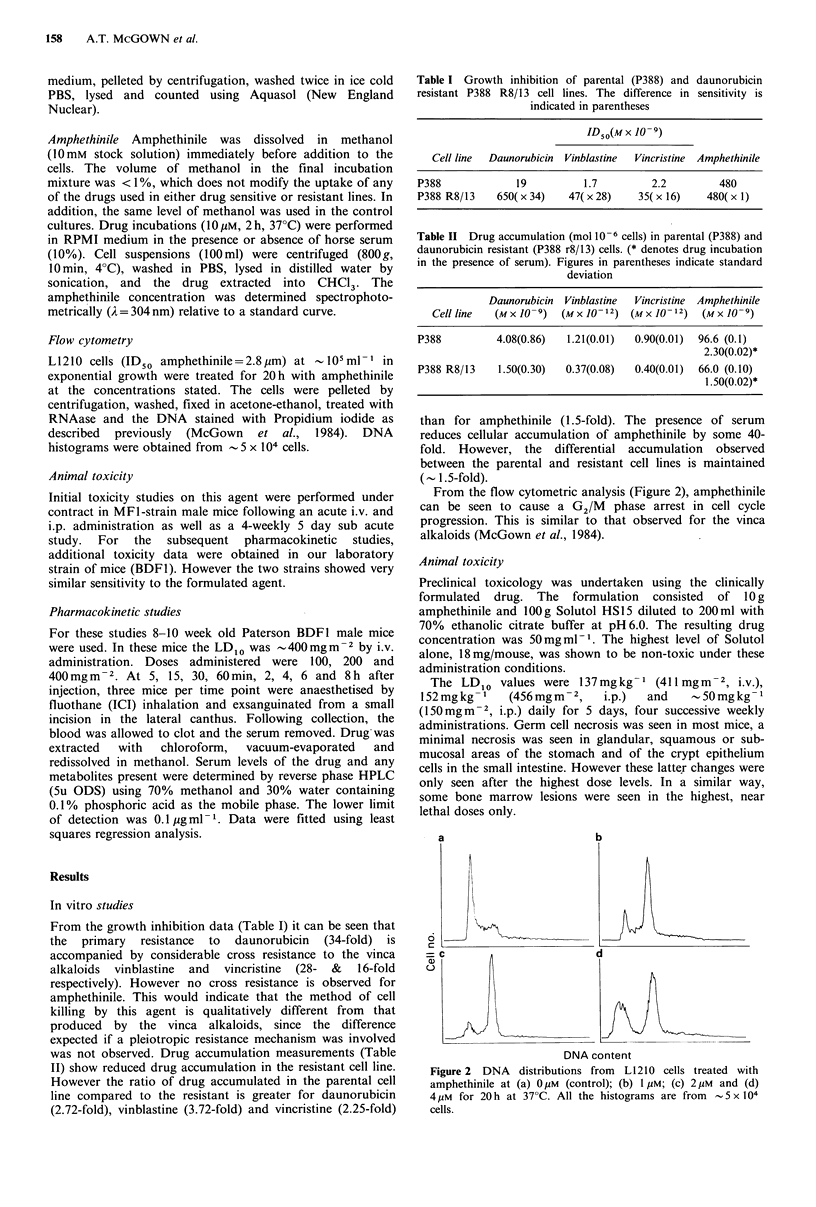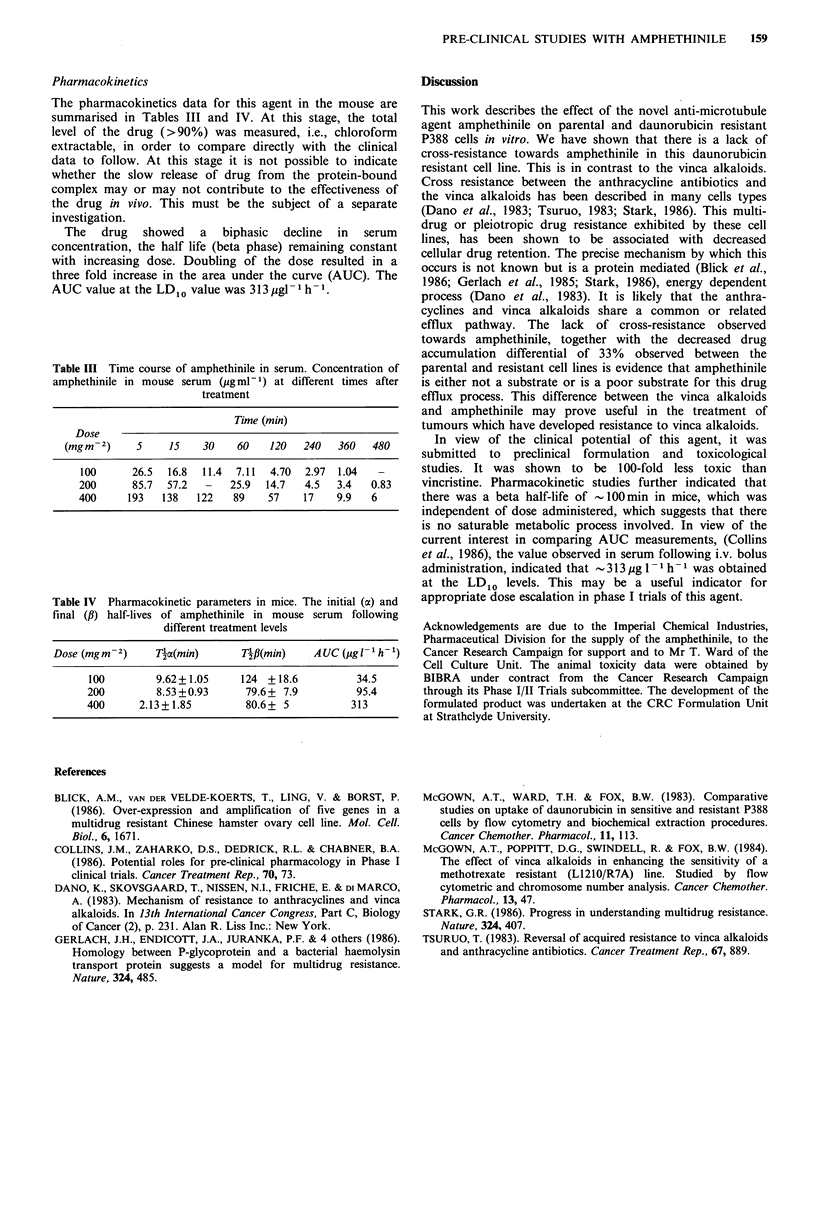# Pre-clinical studies of a novel anti-mitotic agent, amphethinile.

**DOI:** 10.1038/bjc.1988.32

**Published:** 1988-02

**Authors:** A. T. McGown, C. Ewen, D. B. Smith, B. W. Fox

**Affiliations:** Paterson Institute for Cancer Research, Christie Hospital and Holt Radium Institute, Manchester, UK.

## Abstract

A new antitumour agent is described, which has been shown to induce a G2/M block in murine leukaemia cells in vitro. In addition this agent has been shown to be equally toxic toward parental and daunorubicin-resistant P388 cells in vitro. These resistant cells are highly cross-resistant to the established anti-mitotic agents vincristine and vinblastine. Drug accumulation studies in cells have shown that whereas resistance in this cell line is associated with decreased drug accumulation in the case of daunorubicin, vincristine and vinblastine, this effect is much less pronounced for amphethinile. It is proposed that amphethinile is a poor substrate for the drug efflux process associated with the pleiotropic resistance mechanism operating in these cells. The data suggest that cell sensitivity towards amphethinile differs qualitatively from that of the vinca alkaloids and anthracycline. Pharmacokinetic studies in male mice were undertaken. Area under the curve values (AUC), show that levels of approximately 313 micrograms l-1 h-1 were attained at doses equivalent to the LD10. The alpha half life is approximately 8 min after a bolus intravenous injection. The beta half life was approximately 100 min and relatively independent of dose level.


 © The Macmillan Press Ltd., 1988

### Pre-clinical studies of a novel anti-mitotic agent, amphethinile

A.T. McGown, C. Ewen, D.B. Smith & B.W. Fox

*Paterson Institute for Cancer Research, Christie Hospital and Holt Radium Institute, Manchester M20 9BX, UK.*

**Summary** A new antitumour agent is described, which has been shown to induce a $G_2/M$ block in murine leukaemia cells *in vitro*. In addition this agent has been shown to be equally toxic toward parental and daunorubicin-resistant P388 cells *in vitro*. These resistant cells are highly cross-resistant to the established anti-mitotic agents vincristine and vinblastine. Drug accumulation studies in cells have shown that whereas resistance in this cell line is associated with decreased drug accumulation in the case of daunorubicin, vincristine and vinblastine, this effect is much less pronounced for amphethinile.

It is proposed that amphethinile is a poor substrate for the drug efflux process associated with the pleiotropic resistance mechanism operating in these cells. The data suggest that cell sensitivity towards amphethinile differs qualitatively from that of the vinca alkaloids and anthracycline.

Pharmacokinetic studies in male mice were undertaken. Area under the curve values (AUC), show that levels of $\sim 313\,\mu g\,l^{-1}\,h^{-1}$ were attained at doses equivalent to the $LD_{10}$. The alpha half life is ~8 min after a bolus intravenous injection. The beta half life was ~100 min and relatively independent of dose level.

One of the most clinically useful groups of anticancer drugs are the microtubule assembly inhibitors belonging to the vinca alkaloid group. These drugs continue to be extracted and purified from the Catharanthus species as it is still not feasible to synthesise them by any commercially viable procedure. A simple synthetic analogue may therefore provide an alternative spindle poison, with sufficiently different mode of action, cell permeability and other physico-chemical characteristics to be a useful addition to the clinic.

An agent, (2-amino-3-cyano-5-(phenylthio)-indole, ICI 134154, Figure 1) has been identified which appears to affect the microtubular control of mitosis. The compound, named amphethinile, which is currently undergoing clinical trials, has now been studied in more detail to determine if these earlier observations can be substantiated and if so whether the action of the drug closely resembles or differs significantly from that of the vinca alkaloids. It would be interesting to determine if this synthetic compound shows cross resistance to a cell line which exhibits pleiotropic drug response characteristics of some naturally occurring antitumour agents, including the vinca alkaloids (Blick *et al.*, 1986; Dano *et al.*, 1983; Gerlach *et al.*, 1986).

In addition, following perclinical formulation and toxicology, the pharmacokinetic characteristics of this agent have been determined in male mice in preparation for the clinical studies which will be described elsewhere.

### Materials and methods

### *Drugs*

Amphethinile was kindly donated by ICI (Alderley Park, Cheshire, UK). The compound is a pale yellow granular solid, m.p. 190.5–191°C. It is readily soluble in acetone, dimethyl sulphoxide and acetonitrile, but sparingly soluble in toluene. The initial product is >99% pure by spectral, HPLC and TLC analytical methods.

Daunorubicin was obtained from May and Baker (Dagenham, UK) and the vinca alkaloids from Amersham International plc (Amersham, Bucks, UK).

### *Cell culture*

A cell line showing decreased sensitivity to daunorubicin (P388 R8/13) was developed from the parental (P388) cell line by incremental challenge with the drug *in vitro* as previously described (McGown *et al.*, 1983). Both cell lines are grown in RPMI medium supplemented with 10% horse serum (Gibco, UK). Cells are regularly screened and shown to be mycoplasma free. Growth inhibition studies were carried out by back extrapolation of growth curves following a 1 h challenge with drug. Cell counts were performed in triplicate on an electronic cell counter (Coulter Electronics, Luton, UK).

### *Measurement of drug accumulations in cells*

Cells, in exponential growth, were centrifuged (800 *g*, 5 min, 4°C) and resuspended at a cell concentration of $2 \times 10^5\,ml^{-1}$. Drug incubations were performed as described below. Daunorubicin and amphethinile were employed at doses which produced comparable survival data in the resistant cell line. These doses are necessarily high in order to provide for the spectral sensitivity used. Even at this high level however ($20 \times ID_{50}$), the differential accumulation of daunorubicin, characteristic of the pleiotropic drug response to the drug, was evident. The use of the lower levels of radiolabelled vincristine was used to confirm the differential uptake characteristic of the pleiotropic drug resistance. Cell lysis was performed by sonication (MSE 20 μm peak to peak, 20 sec). All experiments were performed in triplicate.

*Daunorubicin* Cells were resuspended in serum-free medium (RPMI) and incubated with daunorubicin (10 μM, 37°C, 2 h). The cells were pelleted by centrifugation (160 *g*, 10 min, 4°C), washed in cold PBS and lysed in distilled water. Daunorubicin was extracted 4 × 3 mls of $CHCl_3$: isoamyl alcohol (24:1) and the concentration determined fluorimetrically ($\lambda$ex = 480 nm, $\lambda$em = 570 nm).

*Vinca alkaloids* Cells were incubated with either $^3H$-vincristine (4.6 Ci mmol$^{-1}$, 25 nm, 1 h, 37°C) or $^3H$-vinblastine (9.9 Ci mmol$^{-1}$, 25 nm, 1 h, 37°C) in serum free

Amphethinile

**Figure 1** Structure of Amphethinile, (ICI 134, 154).

Correspondence: B.W. Fox.
Received 15 June 1987; and in revised form, 7 August 1987.

medium, pelleted by centrifugation, washed twice in ice cold PBS, lysed and counted using Aquasol (New England Nuclear).

*Amphethinile* Amphethinile was dissolved in methanol (10 mM stock solution) immediately before addition to the cells. The volume of methanol in the final incubation mixture was <1%, which does not modify the uptake of any of the drugs used in either drug sensitive or resistant lines. In addition, the same level of methanol was used in the control cultures. Drug incubations (10 $\mu$M, 2 h, 37°C) were performed in RPMI medium in the presence or absence of horse serum (10%). Cell suspensions (100 ml) were centrifuged (800 *g*, 10 min, 4°C), washed in PBS, lysed in distilled water by sonication, and the drug extracted into $CHCl_3$. The amphethinile concentration was determined spectrophotometrically ($\lambda = 304$ nm) relative to a standard curve.

### *Flow cytometry*

L1210 cells ($ID_{50}$ amphethinile = 2.8 $\mu$m) at ~$10^5$ ml$^{-1}$ in exponential growth were treated for 20 h with amphethinile at the concentrations stated. The cells were pelleted by centrifugation, washed, fixed in acetone-ethanol, treated with RNAase and the DNA stained with Propidium iodide as described previously (McGown *et al.*, 1984). DNA histograms were obtained from ~$5 \times 10^4$ cells.

### *Animal toxicity*

Initial toxicity studies on this agent were performed under contract in MF1-strain male mice following an acute i.v. and i.p. administration as well as a 4-weekly 5 day sub acute study. For the subsequent pharmacokinetic studies, additional toxicity data were obtained in our laboratory strain of mice (BDF1). However the two strains showed very similar sensitivity to the formulated agent.

### *Pharmacokinetic studies*

For these studies 8–10 week old Paterson BDF1 male mice were used. In these mice the $LD_{10}$ was ~400 mg m$^{-2}$ by i.v. administration. Doses administered were 100, 200 and 400 mg m$^{-2}$. At 5, 15, 30, 60 min, 2, 4, 6 and 8 h after injection, three mice per time point were anaesthetised by fluothane (ICI) inhalation and exsanguinated from a small incision in the lateral canthus. Following collection, the blood was allowed to clot and the serum removed. Drug was extracted with chloroform, vacuum-evaporated and redissolved in methanol. Serum levels of the drug and any metabolites present were determined by reverse phase HPLC (5u ODS) using 70% methanol and 30% water containing 0.1% phosphoric acid as the mobile phase. The lower limit of detection was 0.1 $\mu$g ml$^{-1}$. Data were fitted using least squares regression analysis.

### Results

### In vitro *studies*

From the growth inhibition data (Table I) it can be seen that the primary resistance to daunorubicin (34-fold) is accompanied by considerable cross resistance to the vinca alkaloids vinblastine and vincristine (28- & 16-fold respectively). However no cross resistance is observed for amphethinile. This would indicate that the method of cell killing by this agent is qualitatively different from that produced by the vinca alkaloids, since the difference expected if a pleiotropic resistance mechanism was involved was not observed. Drug accumulation measurements (Table II) show reduced drug accumulation in the resistant cell line. However the ratio of drug accumulated in the parental cell line compared to the resistant is greater for daunorubicin (2.72-fold), vinblastine (3.72-fold) and vincristine (2.25-fold) than for amphethinile (1.5-fold). The presence of serum reduces cellular accumulation of amphethinile by some 40-fold. However, the differential accumulation observed between the parental and resistant cell lines is maintained (~1.5-fold).

From the flow cytometric analysis (Figure 2), amphethinile can be seen to cause a $G_2$/M phase arrest in cell cycle progression. This is similar to that observed for the vinca alkaloids (McGown *et al.*, 1984).

**Table I** Growth inhibition of parental (P388) and daunorubicin resistant P388 R8/13 cell lines. The difference in sensitivity is indicated in parentheses

| | $ID_{50}$(M × $10^{-9}$) | | | |
|---|---|---|---|---|
| *Cell line* | *Daunorubicin* | *Vinblastine* | *Vincristine* | *Amphethinile* |
| P388 | 19 | 1.7 | 2.2 | 480 |
| P388 R8/13 | 650(×34) | 47(×28) | 35(×16) | 480(×1) |

**Table II** Drug accumulation (mol $10^{-6}$ cells) in parental (P388) and daunorubicin resistant (P388 r8/13) cells. (* denotes drug incubation in the presence of serum). Figures in parentheses indicate standard deviation

| *Cell line* | *Daunorubicin* (M × $10^{-9}$) | *Vinblastine* (M × $10^{-12}$) | *Vincristine* (M × $10^{-12}$) | *Amphethinile* (M × $10^{-9}$) |
|---|---|---|---|---|
| P388 | 4.08(0.86) | 1.21(0.01) | 0.90(0.01) | 96.6 (0.1)<br>2.30(0.02)* |
| P388 R8/13 | 1.50(0.30) | 0.37(0.08) | 0.40(0.01) | 66.0 (0.10)<br>1.50(0.02)* |

### *Animal toxicity*

Preclinical toxicology was undertaken using the clinically formulated drug. The formulation consisted of 10 g amphethinile and 100 g Solutol HS15 diluted to 200 ml with 70% ethanolic citrate buffer at pH 6.0. The resulting drug concentration was 50 mg ml$^{-1}$. The highest level of Solutol alone, 18 mg/mouse, was shown to be non-toxic under these administration conditions.

The $LD_{10}$ values were 137 mg kg$^{-1}$ (411 mg m$^{-2}$, i.v.), 152 mg kg$^{-1}$ (456 mg m$^{-2}$, i.p.) and ~50 mg kg$^{-1}$ (150 mg m$^{-2}$, i.p.) daily for 5 days, four successive weekly administrations. Germ cell necrosis was seen in most mice, a minimal necrosis was seen in glandular, squamous or submucosal areas of the stomach and of the crypt epithelium cells in the small intestine. However these latter changes were only seen after the highest dose levels. In a similar way, some bone marrow lesions were seen in the highest, near lethal doses only.

**Figure 2** DNA distributions from L1210 cells treated with amphethinile at (a) 0 $\mu$M (control); (b) 1 $\mu$M; (c) 2 $\mu$M and (d) 4 $\mu$M for 20 h at 37°C. All the histograms are from ~$5 \times 10^4$ cells.

*Pharmacokinetics*

The pharmacokinetics data for this agent in the mouse are summarised in Tables III and IV. At this stage, the total level of the drug (>90%) was measured, i.e., chloroform extractable, in order to compare directly with the clinical data to follow. At this stage it is not possible to indicate whether the slow release of drug from the protein-bound complex may or may not contribute to the effectiveness of the drug *in vivo*. This must be the subject of a separate investigation.

The drug showed a biphasic decline in serum concentration, the half life (beta phase) remaining constant with increasing dose. Doubling of the dose resulted in a three fold increase in the area under the curve (AUC). The AUC value at the $LD_{10}$ value was 313 $\mu g l^{-1} h^{-1}$.

**Table III** Time course of amphethinile in serum. Concentration of amphethinile in mouse serum ($\mu g ml^{-1}$) at different times after treatment

| *Dose* | *Time (min)* | | | | | | | |
|---|---|---|---|---|---|---|---|---|
| *(mg m$^{-2}$)* | *5* | *15* | *30* | *60* | *120* | *240* | *360* | *480* |
| 100 | 26.5 | 16.8 | 11.4 | 7.11 | 4.70 | 2.97 | 1.04 | – |
| 200 | 85.7 | 57.2 | – | 25.9 | 14.7 | 4.5 | 3.4 | 0.83 |
| 400 | 193 | 138 | 122 | 89 | 57 | 17 | 9.9 | 6 |

**Table IV** Pharmacokinetic parameters in mice. The initial ($\alpha$) and final ($\beta$) half-lives of amphethinile in mouse serum following different treatment levels

| *Dose (mg m$^{-2}$)* | *$T\frac{1}{2}\alpha$(min)* | *$T\frac{1}{2}\beta$(min)* | *AUC ($\mu g l^{-1} h^{-1}$)* |
|---|---|---|---|
| 100 | 9.62 ± 1.05 | 124 ± 18.6 | 34.5 |
| 200 | 8.53 ± 0.93 | 79.6 ± 7.9 | 95.4 |
| 400 | 2.13 ± 1.85 | 80.6 ± 5 | 313 |

### Discussion

This work describes the effect of the novel anti-microtubule agent amphethinile on parental and daunorubicin resistant P388 cells *in vitro*. We have shown that there is a lack of cross-resistance towards amphethinile in this daunorubicin resistant cell line. This is in contrast to the vinca alkaloids. Cross resistance between the anthracycline antibiotics and the vinca alkaloids has been described in many cells types (Dano *et al.*, 1983; Tsuruo, 1983; Stark, 1986). This multi-drug or pleiotropic drug resistance exhibited by these cell lines, has been shown to be associated with decreased cellular drug retention. The precise mechanism by which this occurs is not known but is a protein mediated (Blick *et al.*, 1986; Gerlach *et al.*, 1985; Stark, 1986), energy dependent process (Dano *et al.*, 1983). It is likely that the anthracyclines and vinca alkaloids share a common or related efflux pathway. The lack of cross-resistance observed towards amphethinile, together with the decreased drug accumulation differential of 33% observed between the parental and resistant cell lines is evidence that amphethinile is either not a substrate or is a poor substrate for this drug efflux process. This difference between the vinca alkaloids and amphethinile may prove useful in the treatment of tumours which have developed resistance to vinca alkaloids.

In view of the clinical potential of this agent, it was submitted to preclinical formulation and toxicological studies. It was shown to be 100-fold less toxic than vincristine. Pharmacokinetic studies further indicated that there was a beta half-life of ~100 min in mice, which was independent of dose administered, which suggests that there is no saturable metabolic process involved. In view of the current interest in comparing AUC measurements, (Collins *et al.*, 1986), the value observed in serum following i.v. bolus administration, indicated that ~313 $\mu g l^{-1} h^{-1}$ was obtained at the $LD_{10}$ levels. This may be a useful indicator for appropriate dose escalation in phase I trials of this agent.

Acknowledgements are due to the Imperial Chemical Industries, Pharmaceutical Division for the supply of the amphethinile, to the Cancer Research Campaign for support and to Mr T. Ward of the Cell Culture Unit. The animal toxicity data were obtained by BIBRA under contract from the Cancer Research Campaign through its Phase I/II Trials subcommittee. The development of the formulated product was undertaken at the CRC Formulation Unit at Strathclyde University.

### References

BLICK, A.M., VAN DER VELDE-KOERTS, T., LING, V. & BORST, P. (1986). Over-expression and amplification of five genes in a multidrug resistant Chinese hamster ovary cell line. *Mol. Cell. Biol.*, **6**, 1671.

COLLINS, J.M., ZAHARKO, D.S., DEDRICK, R.L. & CHABNER, B.A. (1986). Potential roles for pre-clinical pharmacology in Phase I clinical trials. *Cancer Treatment Rep.*, **70**, 73.

DANO, K., SKOVSGAARD, T., NISSEN, N.I., FRICHE, E. & DI MARCO, A. (1983). Mechanism of resistance to anthracyclines and vinca alkaloids. In *13th International Cancer Congress*, Part C, Biology of Cancer (2), p. 231. Alan R. Liss Inc.: New York.

GERLACH, J.H., ENDICOTT, J.A., JURANKA, P.F. & 4 others (1986). Homology between P-glycoprotein and a bacterial haemolysin transport protein suggests a model for multidrug resistance. *Nature*, **324**, 485.

McGOWN, A.T., WARD, T.H. & FOX, B.W. (1983). Comparative studies on uptake of daunorubicin in sensitive and resistant P388 cells by flow cytometry and biochemical extraction procedures. *Cancer Chemother. Pharmacol.*, **11**, 113.

McGOWN, A.T., POPPITT, D.G., SWINDELL, R. & FOX, B.W. (1984). The effect of vinca alkaloids in enhancing the sensitivity of a methotrexate resistant (L1210/R7A) line. Studied by flow cytometric and chromosome number analysis. *Cancer Chemother. Pharmacol.*, **13**, 47.

STARK, G.R. (1986). Progress in understanding multidrug resistance. *Nature*, **324**, 407.

TSURUO, T. (1983). Reversal of acquired resistance to vinca alkaloids and anthracycline antibiotics. *Cancer Treatment Rep.*, **67**, 889.